# High Specific Efficiency of Venom of Two Prey-Specialized Spiders

**DOI:** 10.3390/toxins11120687

**Published:** 2019-11-23

**Authors:** Ondřej Michálek, Lucia Kuhn-Nentwig, Stano Pekár

**Affiliations:** 1 Department of Botany and Zoology, Faculty of Science, Masaryk University, Kotlářská 2, 611 37 Brno, Czech Republic; 2 Institute of Ecology and Evolution, University of Bern, Baltzerstrasse 6, CH-3012 Bern, Switzerland; lucia.kuhn@iee.unibe.ch

**Keywords:** LD_50_, toxicity, Araneae, predator-prey interactions, specialization, venom ecological function, ecological niche

## Abstract

The venom of predators should be under strong selection pressure because it is a costly substance and prey may potentially become resistant. Particularly in prey-specialized predators, venom should be selected for its high efficiency against the focal prey. Very effective venom paralysis has been observed in specialized predators, such as spiders preying on dangerous prey. Here, we compared the toxicity of the venoms of two prey-specialized species, araneophagous *Palpimanus* sp. and myrmecophagous *Zodarion nitidum*, and their related generalist species. We injected different venom concentrations into two prey types—the prey preferred by a specialist and an alternative prey—and observed the mortality and the paralysis of the prey within 24 h. We found that the venoms of specialists were far more potent towards the preferred prey than alternative prey. The venoms of generalists were similarly potent towards both prey types. In addition, we tested the efficacy of two venom fractions (smaller and larger than 10 kDa) in araneophagous *Palpimanus* sp. Compounds larger than 10 kDa paralyzed both prey types, but smaller compounds (<10 kDa) were effective only on preferred prey, suggesting the presence of prey-specific compounds in the latter fraction. Our results confirm that prey-specialized spiders possess highly specific venom that allows them to subdue dangerous prey.

## 1. Introduction

Quite a few animals utilize venom in defensive and predatory interactions. Venom systems have evolved independently in different taxa across the whole animal kingdom [1,2,3]. Most animal venoms are highly complex cocktails composed of many bioactive compounds, such as salts, amino acids, and neurotransmitters, but, most notably, proteins and peptides (commonly referred to as toxins) [4]. 

The biochemical composition of venom has been mostly identified in large and medically or pharmacologically important venomous species but has been neglected in many taxa, especially in tiny invertebrates [5]. Thanks to the recent development of new venomic techniques, such as genomics, transcriptomics, and proteomics [6], more and more studies focusing on the venom of neglected species are appearing. The connection between venom composition and its function is particularly interesting from an evolutionary point of view. Both interspecific and intraspecific variation in venom composition [7] has been shown to be driven by many factors, namely age [8,9], season [10], geographic origin [11,12], and sex [13]. But the most prominent selection force in species using venom to catch prey is prey preference [14,15,16].

As the production of such a secretion is metabolically costly [17,18,19,20] and prey can potentially become resistant [21], there should be a strong selection for venom optimization towards a specific prey. Indeed, offensive venoms (i.e., venoms used to immobilize prey) often show higher variation and complexity opposed to defensive venoms, which are more conserved [1,22,23,24]. This suggests that a predator–prey arms race is the driving force behind the evolution of venom complexity. Specific venom toxicity has been found in several lineages of snakes [15,25,26] and prey-specific substances have been identified in a few snakes, cone snails, and spiders [27,28,29]. Coevolution between venom complexity and level of prey specialization has been shown only recently in cone snails and spiders [30,31]. So far, however, specific venom efficacy has rarely been tested in prey-specialized predators. A recent study showed that paralysis efficacy on preferred prey was greater in specialists than in generalists [32].

With more than 48,000 described species [33] with different foraging strategies and diets, spiders represent an ideal model group for the investigation of venom function and composition in the evolutionary context. Spider venoms are rich mixtures of many chemical compounds. Available evidence suggests that small peptides are mainly responsible for the insecticidal activity of the venom [34]. The complexity of both smaller (2–15 kDa) and larger components (15–250 kDa) is less diverse in the venom of specialized spiders compared to generalists [31], but it is not known which components are responsible for the efficiency of their venom. 

**Figure 1:**
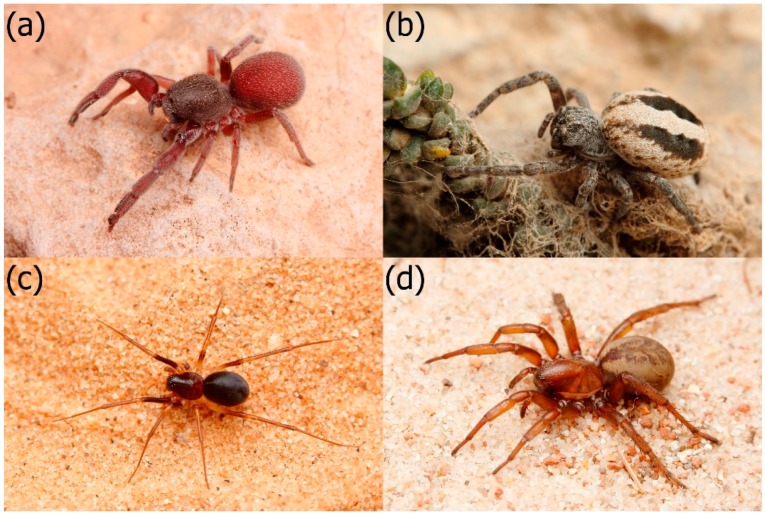
Studied spiders: (**a**) The spider-eating specialist *Palpimanus* sp., (**b**) its related generalist *Stegodyphus lineatus*, (**c**) the ant-eating specialist *Zodarion nitidum*, and (**d**) its related generalist *Cybaeodamus taim*. Photos: O. Michálek.

In this study, we investigated the venom toxicity of one spider-eating (araneophagous) specialist, *Palpimanus* sp. (Figure 1a); one ant-eating (myrmecophagous) specialist, *Zodarion nitidum* (Audouin, 1826) (Figure 1c); and two generalist species, *Stegodyphus lineatus* (Latreille, 1817) (Figure 1b) and *Cybaeodamus taim* Lise, Ott & Rodrigues, 2009 (Figure 1d), to preferred and other prey using laboratory bioassays. We predicted that venom of specialists should be highly potent with respect to preferred prey. Then we compared the toxicity of two venom fractions in the spider-eating species to reveal which venom fraction was responsible for the incapacitation of two distinct prey types.

## 2. Results

### 2.1. Efficiency of Crude Venoms

#### 2.1.1. Mortality

**Figure 2:**
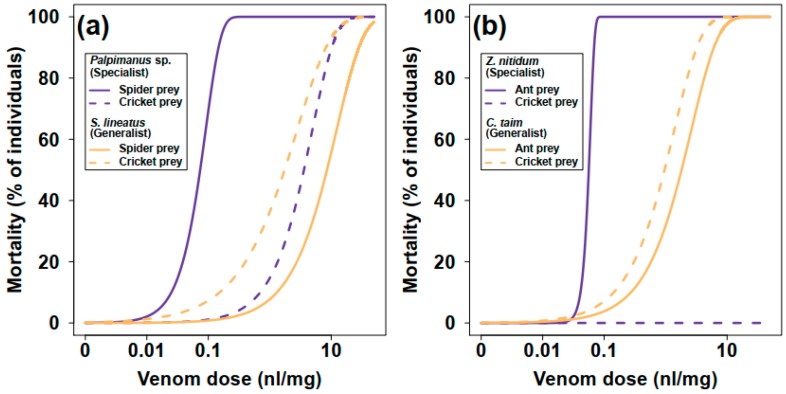
Estimated dose-response models showing the relationship between prey mortality and venom dose for (**a**) spider-eating and (**b**) ant-eating specialists and their related generalists after 24 h from the injection.

**Figure 3:**
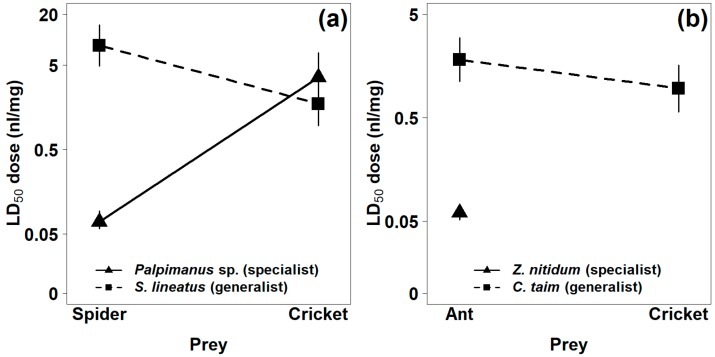
Comparison of estimated median lethal doses (LD_50_) of the venoms of (**a**) spider-eating and (**b**) ant-eating specialists and their related generalists for two prey types 24 h after venom injection. LD_50_ of *Z. nitidum* venom on cricket prey is not shown, as there was no effect. Vertical lines represent 95% confidence intervals.

The mortality of prey within 24 h after venom injection was used as a measure of venom potency. Mortality increased with increasing venom dose. The venom of the araneophagous specialist (*Palpimanus* sp.) was 50 times more potent on spiders than on crickets (GLM-b, χ^2^_1_ = 82.4, *p* < 0.0001), while the venom of the related generalist (*S. lineatus*) was five times more potent on crickets than on spiders (GLM-b, χ^2^_1_ = 14.2, *p* < 0.001, Table 1, Figure 2a and Figure 3a). The venom of the myrmecophagous specialist (*Z. nitidum*) was very potent on ant prey but not on crickets (GLM-b, χ^2^_1_ = 94.6, *p* < 0.0001), while the venom of the related generalist (*C. taim*) was similarly potent on crickets and ants (GLM-b, χ^2^_1_ = 2.9, *p* = 0.09, Table 1, Figure 2b and Figure 3b). The interaction between degree of specialization and prey type was significant for both specialist–generalist pairs—for *Palpimanus* sp. and *S. lineatus* (GLM-b, χ^2^_1_ = 73.0, *p* < 0.0001; Figure 3a) and also for *Z. nitidum* and *C. taim* (GLM-b, χ^2^_1_ = 85.0, *p* < 0.0001; Figure 3b)—suggesting the venoms of specialists are prey-specific.

#### 2.1.2. Paralysis

The proportion of prey paralyzed within one hour of venom injection was used to estimate the paralyzing properties of the venoms. The paralyzing dose of *Palpimanus* sp. venom was more than 10 times lower for spider prey than for cricket prey (GLM-b, χ^2^_1_ = 86.8, *p* < 0.0001), while the paralyzing dose of *S. lineatus* was more than 30 times higher for spider prey than for cricket prey (GLM-b, χ^2^_1_ = 58.3, *p* < 0.0001, Table 1). The venom of *Z. nitidum* caused paralysis in ant prey but had no effect on crickets (GLM-b, χ^2^_1_ = 53.1, *p* < 0.0001), while the paralyzing dose of *C. taim* venom was similar for crickets and ants (GLM-b, χ^2^_1_ = 0.7, *p* = 0.39, Table 1). The interaction between degree of specialization and prey type was also significant for both pairs (*Palpimanus* sp. and *S. lineatus*: GLM-b, χ^2^_1_ = 40.5, *p* < 0.0001; *Z. nitidum* and *C. taim*: GLM-b, χ^2^_1_ = 40.9, *p* < 0.0001). These results show that the venoms of the two prey-specialized spiders were far more efficient on their preferred prey (spider or ant) and less efficient or even inefficient on alternative prey (cricket). The venom of the generalists was similarly potent on both prey types or more potent on the alternative prey.

**Figure 4:**
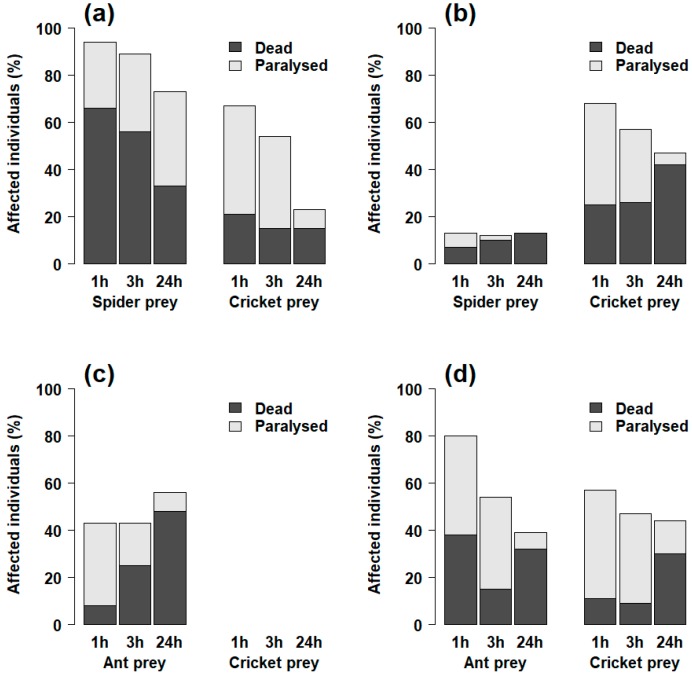
Comparison of the proportions of dead or paralyzed prey individuals 24 h after injection with various concentrations of venom (see Table S1) from (**a**) *Palpimanus* sp. (spider-eating specialist), (**b**) *S. lineatus* (generalist), (**c**) *Z. nitidum* (ant-eating specialist), and (**d**) *C. taim* (generalist).

In addition, we compared the paralyzing and lethal effects of the venoms. The paralyzing effect of *Palpimanus* sp. venom was even stronger than the lethal effect on both spiders and crickets (GEE-b, χ^2^_1_ = 133.9, *p* < 0.0001, Table 1). It was, however, at least partially reversible, as some individuals injected with lower venom concentrations were able to recover after three and 24 h (Figure 4a). In *S. lineatus*, paralysis after one hour did not differ from mortality after 24 h for spiders (GEE-b, χ^2^_1_ = 0.0, *p* = 0.99), but was more prominent for crickets (GEE-b, χ^2^_1_ = 10.2, *p* < 0.01, Table 1, Figure 4b). Although the effective (paralyzing) dose of *Z. nitidum* venom did not differ significantly from the lethal dose (GEE-b, χ^2^_1_ = 2.8, *p* = 0.09, Table 1), *Z. nitidum* venom caused increasing mortality in ants over time (GEE-b, χ^2^_1_ = 20.9, *p* < 0.0001, Figure 4c). The effective dose of *C. taim* venom was lower than the lethal dose for both spiders and crickets (GEE-b, χ^2^_1_ = 44.1, *p* < 0.0001, Table 1, Figure 4d). 

### 2.2. Efficiency of Venom Fractions

**Figure 5:**
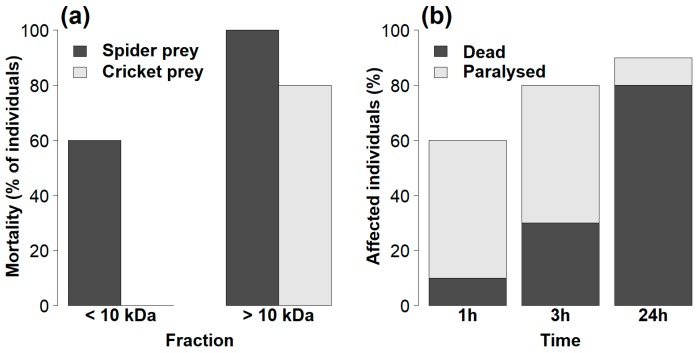
(**a**) Comparison of the toxicities of the venom fractions of *Palpimanus* sp. towards two prey types 24 h after venom injection. (**b**) Effects of high mass compounds (>10 kDa) of *Palpimanus* sp. venom on cricket prey over 24 h.

The toxicity of the venom fractions of spider-eating *Palpimanus* sp. differed between both fractions (GLM-b, χ^2^_1_ = 15.6, *p* < 0.0001) and prey types (GLM-b, χ^2^_1_ = 13.4, *p* < 0.001). Low mass compounds (<10 kDa) caused 60% morality in spiders only, while high mass compounds (>10 kDa) caused 100% and 80% mortality in both spiders and crickets, respectively (Figure 5a). The effect of venom dose (after taking into account prey mass) was not significant (GLM-b, χ^2^_1_ = 3.0, *p* = 0.09). The mortality was similar after 1, 3, and 24 h for both combinations of fractions and prey, except for high mass compounds injected into cricket prey, where increasing mortality over time was observed (GEE-b, χ^2^_1_ = 9.2, *p* < 0.01, Figure 5b).

## 3. Discussion

Prey-specialized spiders often hunt prey that can be potentially harmful due to the possession of effective defenses, such as venom in other spiders, mandibles and stings in ants, and mandibles in termites. Therefore, there should be a strong selection for quickly subduing such prey to prevent retaliation. This can be achieved either through venom or silk optimization, the two primary weapons of spiders used in prey capture [35].

In a previous study in which prey was offered to a predator and the time to paralysis was observed, we found that venoms of prey-specialized spiders induced shorter paralysis latency in preferred prey than in alternative prey [32], suggesting the functional adaptation of venom to a certain prey. However, the observed paralysis latency could be affected by several other factors, such as the venom dose, the site of the bite, or the activity of the prey, which were not controlled for. Indeed, venom metering—the ability to optimize the use of venom—has been documented in snakes, scorpions, and also spiders [2]. Spiders can increase the amount of injected venom when a prey is resisting [36] or when a dangerous prey is attacked [37]. The faster prey immobilization we observed in a previous study [32] could have been caused by the use of a larger amount of venom rather than by greater specific toxicity. To verify the hypothesis of venom specificity resulting from specific venom compositions, a different approach must be taken, such as the venom bioassays performed in this study. 

Comparative studies of venom efficiency between related venomous species are still scarce and usually performed on one prey type only. Here, we used two prey types and revealed significant differences in venom toxicities between related spider specialists and generalists. We confirmed that venoms of specialists are indeed more potent towards their preferred prey than alternative prey, suggesting that these venoms are primarily tailored to affect the target prey of these predators. 

Prey-specialized spiders are not strictly monophagous, with one exception [38], but they specialize on several species from a higher taxon (e.g., order) [39]. Thus, we used only one prey species representing the focal prey for each specialist. Nevertheless, *Palpimanus* spiders can effectively subdue a wide range of spider species from different families and guilds [40], suggesting that its venom is specifically effective towards spiders. Similarly, *Z. nitidum* captured several ant species in laboratory experiments [41], although the paralysis latency was shorter for Formicinae compared to Myrmicinae. Indeed, some *Zodarion* spiders specialize on one of the ant subfamilies [42]. To verify whether the venoms of *Zodarion* spiders are specific to ant subfamilies, bioassays with different ant species would need to be performed.

In our experiments, we used prey model species that represent non-native surrogate prey of the studied spiders because natural prey species would have been difficult to obtain in sufficient numbers for the bioassays. The predator–prey arms race may lead to the reduced susceptibility of sympatric prey [43]. Venom toxicity may therefore differ for native and related non-native prey [44]. However, it seems that even the natural prey of prey-specialized spiders are highly susceptible to their venom [32,45].

Specialized spiders are rare, and they usually possess relatively small venom glands [31], which makes obtaining a sufficient amount of venom challenging. Even though we pooled the venom from several individuals, it was still not enough to perform many replicates of the bioassays. Nevertheless, the pattern was always similar for the venoms of both tested species pairs—venoms of specialized spiders were more potent towards their preferred prey than alternative prey, while venoms of generalized spiders were similarly potent to both prey types or more potent towards alternative prey.

The venom of *Z. nitidum* was not at all effective towards crickets. Because of the extremely small volume of crude venom obtained, we were able to use only lower concentrations of venom, which were effective towards ants but not towards crickets. It has been shown that *Acheta domesticus* crickets are less sensitive to the venoms of some spiders (e.g., *Cupiennius salei* (Keyserling, 1877)) [46]. It is still unknown whether higher concentrations of *Z. nitidum* venom would have some effect on crickets or not. Nevertheless, the effectivity on crickets would still be lower. In addition, *Z. nitidum* possesses tiny venom glands [31] and therefore probably uses a low amount of venom during prey capture (which is equivalent to a lower venom concentration). *Z. nitidum* is capable of paralyzing some alternative prey, like termites, but the paralysis latency is longer than for ants [32]. Due to the strict specialization of spiders of the genus *Zodarion* on ant prey [47], the expression of ant-specific toxins may be optimized which, in turn, results in less efficient paralysis for other prey types. 

It has been shown that different spider toxins may vary in their paralytic effects [48]. We observed such divergence in effects of the whole venom between both specialized spiders. *Palpimanus* sp. venom induced a strong immediate paralysis that was reversible at lower doses (although recovery after three or 24 h is probably not ecologically relevant, as a spider would feed on each item that no longer moves after a few minutes), while *Z. nitidum* venom caused irreversible mortality which increased in time. Differences in hunting tactics can explain such different venom action. *Palpimanus* grabs the prey (i.e., spiders) and holds it tightly with its forelegs for a period of several minutes until the prey is immobilized [40]. As the prey is dangerous (during contact), it could attack *Palpimanus*. It is therefore essential that the venom rapidly immobilizes the prey. On the other hand, *Zodarion* spiders first quickly bite an ant, then release it and wait at a safe distance until the ant is paralyzed [49]. Due to the absence of longer direct contact with the prey, unlike in *Palpimanus*, the venom must prevent prey escape by causing gradual mortality. Not surprisingly, the paralysis latency of preferred prey is shorter in *Palpimanus* compared to *Z. nitidum* [32]. Therefore, the observed venomic activities of these spiders are complementary with their behavioral prey capture adaptations.

In many spiders, small peptides (<10 kDa) are responsible for venom paralytic activity [34], while in some others large proteins are effective [50]. To find which compounds are effective in prey specialized spiders, we split the whole venom into two fractions. In the case of *Z. nitidum*, the obtained volume was too small to perform bioassays with fractions. The two venom fractions of *Palpimanus* venom had different effects on two prey types. Both fractions had a paralyzing/lethal effect on spider prey, but only the fraction of high molecular compounds (>10 kDa) was effective on crickets. However, the unfractionated venom may be more ecologically functional or potent, as venom components can act synergistically [34]. 

The identity of toxins and their mode of action in these two specialized spider species are entirely unknown. The most common toxins responsible for prey incapacitation discovered so far in spiders are neurotoxins, especially disulfide-rich peptide neurotoxins, but also larger compounds like the latrotoxins of widow spiders [34]. Recently, compounds with specific activities other than neurotoxins have been discovered, like linear cytolytic polypeptides in the venom of the zodariid spider *Lachesana tarabaevi* (Zonstein & Ovtchinnikov, 1999) [51]. We hope to identify the compounds in the future once the proteomic and transcriptomic data for the venom of these two species are available.

Unfortunately, the research on spider venom was biased towards only several spider taxa [52]. The diversity of the different toxin families among spiders may be much higher than anticipated, as has been shown for other venomous taxa. For example, even in closely related snakes specialized on mammals, different toxins are expressed to incapacitate the prey [53]. A recent study on centipedes revealed that the venom composition differs markedly among five chilopod orders [54]. Our recent comparative study on several specialized spiders [32] revealed that phylogenetically unrelated spider species specialized on similar prey (e.g., anteaters) might have different venom compositions. This suggests that the same target (i.e., paralysis of the same type of prey) is achieved in a variety of ways. The venom investigation of more prey-specialized spiders is, therefore, much needed to unveil the true diversity of spider toxins with a similar effect.

In recent years, demand for the development of ecofriendly biopesticides that would replace conventional chemical pesticides has arisen [55]. In this regard, spider venoms represent an ideal source of potential bioinsecticides [34]. As we show here, the venom of prey-specialized *Zodarion* spiders is especially effective towards ant prey. Ants are one of the most prominent terrestrial animals in terms of biomass [56] and are also important pest species in several regions [57]. Research focused on the venom of prey-specialized spiders could provide new bioactive compounds that could be potentially useful in the development of biopesticides.

## 4. Materials and Methods 

### 4.1. Materials

We used four spider species and three prey species. As a spider-eating specialist, we used *Palpimanus* sp. (Palpimanidae, *n* = 9, Figure 1a) collected in the Ndumo Game Reserve in South Africa. *Palpimanus* spiders prefer spider prey, but also capture alternative prey (including crickets) at a lower frequency in the laboratory [40]. As an ant-eating specialist, we used *Zodarion nitidum* (Audouin, 1826) (Zodariidae, *n* = 8, Figure 1c) collected in the Negev desert in Israel. *Z. nitidum* is strictly specialized on ants but prefers Myrmicinae ants as prey [41]. As generalists phylogenetically related to the specialists, we used *Stegodyphus lineatus* (Latreille, 1817) (Eresidae, *n* = 5, Figure 1b) collected in the Negev Desert in Israel and *Cybaeodamus taim* Lise, Ott & Rodrigues, 2009 (Zodariidae, *n* = 5, Figure 1d) collected in Uruguay. We used *S. lineatus,* a generalist from the closely related family Eresidae, as the whole family Palpimanidae is considered araneophagous [39]. *S. lineatus* is a web-building species that captures mainly flying insects (such as hymenopterans, flies, and beetles), but also orthopterans and other prey [58]. *C. taim* accepted a variety of prey in the laboratory, including spiders, ants, and crickets [59]. Spiders were kept singly in glass tubes (height: 6 cm, diameter: 1.5 cm) with moisturized gypsum on the bottom, in a chamber at room temperature (22 °C) and under a 16:8 h light to dark regime. *Palpimanus* spiders were fed regularly with other spiders, *Z. nitidum* with ants, and the two spider generalists with a mixed diet (spiders/ants and crickets). Water was provided every three days.

As prey, we used juvenile *Pardosa* sp. spiders (*n* = 235, body mass: 9.09 ± 2.79 mg), which were used for the injection of venom of *Palpimanus* and *S. lineatus*; *Lasius flavus* (Fabricius, 1782) ant imagoes (*n* = 133, body mass: 0.94 ± 0.35 mg), which were used for the injection of the venom of *Z. nitidum* and *C. taim*; and *Acheta domesticus* (Linnaeus, 1758) juvenile crickets (*n* = 211, body mass: 5.60 ± 4.39 mg), which were used for the injection of the venom of all four spider species. *Pardosa* spiders and *L. flavus* ants were collected in the surroundings of the Department of Botany and Zoology, Masaryk University, Brno. *Pardosa* spiders were kept singly in punctured Eppendorf tubes placed in a plastic bag with moisturized cotton and *L. flavus* ants were kept together in a plastic box filled with soil. Both the spiders and the ants were kept in a chamber at low temperature (10 °C) and under a 16:8 h light to dark photoperiod. Crickets were bought at a local pet store. No permission was needed to use the animals.

### 4.2. Obtaining the Venom

Venom was obtained from spiders by means of an electrical milking technique [60,61,62]. A spider was anesthetized with CO_2_ for 2 min, placed on a stub and covered with a mesh, and the venom was collected in a glass microcapillary (volume 0.5 or 1 µl) that was slid onto one of the fangs of the spider’s chelicerae. The spider was teased with an electric impulse and released venom into the capillary. Individual spiders were milked several times in approximately three-week intervals. Micro capillaries containing the venom were stored in the freezer at −20 °C before further processing.

### 4.3. Bioassays with Crude Venom

The crude venom obtained from each species was pooled, and venom samples of different concentrations were prepared for the venom toxicity bioassays [62]. Ammonium acetate buffer (0.1M, pH = 6.11) was used to dissolve venom samples. Each tested prey individual was weighed using a Kern 770 balance (Balingen, Germany) with a precision of 0.01 mg, so the precise venom dose in nL/mg of each individual could be calculated. Prey was anesthetized with CO_2_ before injection. Then, 50 nL of the diluted venom sample was injected into the thorax or prosoma of the tested prey using a calibrated glass microsyringe. Several venom samples of different concentrations that caused a dose/weight-dependent effect were used. Approximately 10 to 20 prey individuals were used per each sample (Table S1). Simultaneously, only ammonium acetate buffer was injected into 10 prey individuals as a control to exclude the effect of merely piercing the prey on the mortality of the prey. If there was mortality in the control group, the data for the given trial was discarded. After injection, the prey was placed individually into small Petri dishes (diameter 35 mm) with a small piece of moisturized cotton. The mortality of the prey was checked 24 h after injection. In addition, the prey was observed one and three hours after injection. Prey was considered dead or completely paralyzed when there was no movement after a light touch with a pincer and paralyzed when it was unable to move in the Petri dish normally (it was not able to walk and/or moved erratically, etc.). 

### 4.4. Bioassays with Venom Fractions of Palpimanus sp.

The crude venom of *Palpimanus* sp. was separated into two fractions. The crude venom sample (2 µl) was diluted in 100 µl of 50 mM PBS and then applied on a 10 kDa centrifugal filter unit Microcon-10 (Merck Millipore, Darmstadt, Germany). The low mass fraction (<10 kDa) was obtained by centrifugation at 14,000 g. The high mass fraction (>10 kDa), which remained on the upper part of the filter, was collected after being shaken in an additional 25 µl of 50 mM PBS.

Both fractions were diluted in ammonium acetate buffer, and one concentration for the given prey was prepared (1:50 for spider prey, corresponding to a venom dose of 0.43 ± 0.16 nL/mg; 1:10 for cricket prey, corresponding to a venom dose of 0.73 ± 0.14 nL/mg). This concentration was higher than the median lethal dose value from the previous experiment. Therefore, it was expected to induce paralysis or death in prey. The diluted venom fractions were injected into 10 individuals of preferred and alternative prey types (spider and cricket) in the same manner as in the bioassay with crude venom, and paralysis/mortality was checked after 24 h, with additional observations after one and three hours. As a control, only a buffer was injected into 10 prey individuals.

### 4.5. Data Analysis

Venom toxicities were compared using dose-response analyses (Figure 2) performed in the R environment [63]. A complementary log-log model with binomial distribution using generalized linear models (GLM-b) was used. The mortality of the prey after 24 h was the response variable, log-transformed venom dose (in nL per mg) was a covariate, and venom origin and prey type were factors. The effect of the venoms on different prey was compared in separate models for each specialist–generalist pair. Median lethal dose values (LD_50_) within 24 h for each combination of venom and prey type were estimated from models using the dose.p function from the MASS package [64]. A 95% confidence interval for each LD_50_ value was calculated using the formula for normal distribution [65]. 

To evaluate the paralyzing properties of each venom, effective doses (ED_50_) were estimated from the models (GLM-b), where the affected prey (dead or paralyzed) after one hour was used as the response variable instead of mortality. Comparison between the paralyzing and lethal effect for each spider was made using another model with the type of effect (paralysis/mortality) as another factor. In the latter case, generalized estimating equations with binomial distribution (GEE-b) from the geepack package [66] were used instead of GLM-b, as the rate of affected prey after one and 24 h represents repeated measurements on prey individuals. An autoregressive correlation structure (AR1) for replicated observations over time was used to account for these temporal replications [67].

The toxicity of *Palpimanus* sp. venom fractions was also compared using a generalized linear model with a binomial distribution (GLM-b). The mortality of the prey after 24 h was the response variable, venom concentration (in nL/mg) was a covariate, and venom fraction and prey type were factors. GEE-b was used to compare differences in mortality over 24 h. 

## Figures and Tables

**Table 1:** Median lethal (LD_50_) and median effective (paralyzing, ED_50_) doses of crude venoms on two prey types. Numbers in brackets represent 95% confidence intervals. Lethal doses were estimated from the mortality within 24 h after venom injection. Effective doses were calculated according to paralysis occurring one hour after injection.

Spider Species	Preferred Prey (Spider/Ant)		Alternative Prey (Cricket)	
	LD_50_ (nL/mg)	ED_50_ (nL/mg)	LD_50_ (nL/mg)	ED_50_ (nL/mg)
*Palpimanus* sp. (specialist)	0.07 (0.06, 0.09)	0.01 (0.00, 0.02)	3.60 (1.84, 7.02)	0.13 (0.01, 0.17)
*Stegodyphus lineatus* (generalist)	8.52 (4.86, 14.95)	8.66 (4.82, 15.54)	1.74 (0.96, 3.17)	0.27 (0.14, 0.53)
*Zodarion nitidum* (specialist)	0.06 (0.05, 0.06)	0.07 (0.06, 0.08)	no effect	no effect
*Cybaeodamus taim* (generalist)	1.82 (1.11, 2.96)	0.17 (0.07, 0.40)	0.96 (0.57, 1.62)	0.25 (0.15, 0.44)

## Supplementary Materials

The following are available online at https://www.mdpi.com/2072-6651/11/12/687/s1, Table S1: Number of prey (spider/ant and cricket) individuals injected with different concentrations (venom:buffer dilution ratios) of the crude venom of four spiders.
