# Supplementary material for: High Specific Efficiency of Venom of Two Prey-Specialized Spiders
Source: Toxins (Basel). 2019 Nov 23;11(12):687. doi: 10.3390/toxins11120687 (PMC6950493; doi:10.3390/toxins11120687)
Supplement: Supplementary file 1 [file toxins-11-00687-s001.pdf]

Supplementary Materials: High Specific Efficiency of Venom of Two Prey-Specialized Spiders

Ondřej Michálek, Lucia Kuhn-Nentwig and Stano Pekár

Table S1. Number of prey (spider/ant and cricket) individuals injected with different concentrations (venom:buffer dilution ratios) of the crude venom of four spiders.

Concentration (Venom:Buffer Dilution Ratio)	No. of Injected Prey							
	Palpimanus sp.		*S. lineatus*		*Z. nitidum*		*C. taim*	
	Spider	Cricket	Spider	Cricket	Ant	Cricket	Ant	Cricket
1:1200					20			
1:1000					10			
1:900					20			
1:800					17			
1:600	10		10					
1:500	10		10					
1:400	20		10		10		9	
1:300	20		10					
1:200	20	8	10				8	
1:100	20	10	10	10		10	19	18
1:50	20	20		20		10	19	17
1:10	16	20	10	20		10	9	20
pure venom		3	29	15				
**TOTAL**	**136**	**61**	**99**	**65**	**77**	**30**	**64**	**55**
